# EZH2 mitigates the cardioprotective effects of mesenchymal stem cell-secreted exosomes against infarction via HMGA2-mediated PI3K/AKT signaling

**DOI:** 10.1186/s12872-022-02533-9

**Published:** 2022-03-09

**Authors:** Wei Jiao, Jie Hao, Yanan Xie, Mingjie Meng, Weinian Gao

**Affiliations:** 1 Department of Cardiology, The Second Hospital of Hebei Medical University, Shijiazhuang, 050000 Hebei People’s Republic of China; 2 Department of Cardiac Macrovascular Surgery, The Second Hospital of Hebei Medical University, No. 215, Heping West Road, Xinhua District, Shijiazhuang, 050000 Hebei People’s Republic of China

**Keywords:** Mesenchymal stem cell-derived exosomes, EZH2, HMGA2, PI3K/AKT pathway, Myocardial fibrosis

## Abstract

**Background:**

Mesenchymal stem cell-derived exosomes (MSC-EXO) have emerged as novel therapeutic strategies for myocardial infarction (MI). However, many questions remain untouched and unanswered regarding their roles in myocardial fibrosis. This study aimed to probe the therapeutic effects of MSC-EXO on myocardial fibrosis after MI and possible mechanisms.

**Methods:**

Myocardial tissues were obtained from MI rats, and myocardial cell viability, fibrosis, apoptosis, and epithelial–mesenchymal transition (EMT) were detected by immunohistochemistry, Masson’s staining, TUNEL, and western blot. Bone marrow-derived MSCs and corresponding EXO were identified, and cardiac function were detected after treatment of MSC-EXO. Bioinformatics analysis and ChIP assay were conducted to detect the downstream genes of EZH2. EZH2 was upregulated alone or with HMGA2 overexpression in myocardial tissues of MI rats upon MSC-EXO treatment, and PI3K/AKT pathway activity in myocardial tissues was detected using western blot.

**Results:**

The proliferative activity in myocardial tissues of MI rats was significantly decreased, along with accentuated fibrosis, increased collagen volume and EMT. MSC-EXO treatment resulted in partial restoration of cardiac function and reduced EZH2 expression in the myocardium of rats. EZH2 inhibited HMGA2 expression by increasing the H3K27me3 modification. PI3K/AKT pathway was altered under the influence of the EZH2/HMGA2 axis. EZH2 inhibited the effect of MSC-EXO on the recovery of cardiac function and accelerated fibrosis, while HMGA2 reversed the effect of EZH2 to reduce fibrosis and enhance cardiac function.

**Conclusion:**

MSC-EXO alleviated fibrosis in MI rats via inhibition of EZH2, whereas EZH2 inhibited HMGA2 expression and impaired the PI3K/AKT pathway.

**Supplementary Information:**

The online version contains supplementary material available at 10.1186/s12872-022-02533-9.

## Background

Myocardial infarction (MI) contributes to a substantial loss of functional cardiomyocytes, which is a main cause of mortality worldwide [1]. In nearly all cardiac diseases, fibrosis occurs, mostly consisting of collagen I, and replacement fibrosis ensues cardiomyocyte loss in MI [2]. Epithelial–mesenchymal transition (EMT), an embryonic program relaunched during pathological conditions such as fibrosis, is defined as a cellular process through which cells lose their epithelial identity and acquire a mesenchymal phenotype [3]. As a consequence, understanding the potential molecular mechanisms that underlie EMT and fibrosis will aid in the development of innovative therapies against MI.

Mesenchymal stem cells (MSC), a type of prototypic stem cells with capacity for self-renewal and differentiation with a wide tissue distribution, have been described to improve left ventricular (LV) function, induce reverse remodeling, and decrease scar size [4]. Moreover, bone marrow-derived MSC attenuate myocardial ischemia–reperfusion injury in mice [5]. The paracrine functions of MSCs have been suggested to be mediated by extracellular vesicles, including exosomes (EXO) and microvesicles [6]. EXO, the most common subtype, are nanosized membrane particles that are secreted by cells and can transmit information from cell to cell via their protein and RNA payloads [7]. In addition to eliciting a prospective source of cardiovascular biomarkers, EXO may offer a nonimmunogenic approach of controlling the heart [8]. For instance, human umbilical vein endothelial cells-derived EXO overexpressing HIF-1α exerted cardioprotective effects on rat MI via reducing fibrosis [9]. In the present study, bone marrow-derived MSC were used as the source of EXO (MSC-EXO) because bone morrow is the most common clinical source of adult MSC [10]. Intriguingly, amnion-derived MSC-EXO promoted the survival of trophoblasts under hypoxia by the downregulation of enhancer of zeste 2 polycomb repressive complex 2 subunit (EZH2) [11]. Moreover, EZH2 activation has been reported to contribute to renal EMT and fibrosis through activating multiple signaling pathways, which suggests that EZH2 might be a therapeutic target for treatment of renal fibrosis [12]. However, its function in MI has not been clearly established. Therefore, we postulated that MSC-EXO alleviate fibrosis following MI via inhibiting EZH2 as well. Since EZH2 catalyzes the methylation of lysine 27 of histone H3 (H3K27me), a modification associated with gene repression [13], we hypothesized that EZH2 mediated by MSC-EXO plays a regulatory role in MI by acting as a methyltransferase. In this study, we intended to probe the therapeutic effects of MSC-EXO (MSC-EXO) on MI and to explore the potential epigenetic mechanisms.

## Methods

### Ethical approval

All animals have received human care. All animal procedures were performed in accordance with the Guide for the Care and Use of Laboratory Animals, published by the US National Institutes of Health (NIH Publication No. 85-23, revised in 1996) and approved by the Animal Ethics Committee of the Second Hospital of Hebei Medical University and conform to the ARRIVE guidelines.

### Animals

Male Sprague–Dawley rats (6–8 weeks old) exhibiting normal grooming and walking behavior were purchased from Charles River (Wilmington, MA, USA) and selected for surgery. Anesthesia with 1% sodium pentobarbital (90 mg/kg) was conducted by an intraperitoneal injection after 7 days of adaptive feeding. After the rats were fully anesthetized and fixed, they were routinely shaved and disinfected. The skin of the neck was incised to expose the trachea for ventilation. After successful ventilation, the skin was incised between the 3rd and 4th ribs at the left edge of the sternum to fully expose the heart. The pericardium was cut to find the left coronary vein between the arterial cone and the root of the left auricle, which was used as a marker to ligate the left anterior descending (LAD) about 3–4 cm below the left auricle with a thread between the pulmonary cone causing acute MI. Dimethylsulfoxide (DMSO) or 100 μg MSC-EXO was administrated into rats through the tail vein for 7 consecutive days, 1 week before LAD ligation. Serotype 9 adeno-associated viruses (6 × 10^10^ vector genomes per rat) harboring EZH2-negative control (NC), EZH2-OE, EZH2-OE + HMGA2-NC, EZH2-OE + HMGA2-OE (Hanbio, Shanghai, China) were injected into the myocardium in the peri-infarct region at the time of LAD ligation [14].

### Cardiac function assessment

After complete anesthesia with an intraperitoneal injection of 1% pentobarbital sodium at 80 mg/kg, the LV end-systolic diameter (LVESV), LV end-diastolic diameter (LVEDV), left ventricular end-diastolic internal diameter (Dd) and end-systolic internal diameter (Sd) were measured using a small animal ultrasound imaging system (VisualSonics Inc., Toronto, Canada). LV ejection fraction (LVEF) was calculated as = (LVEDV − LVESV)/LVEDV × 100% and left ventricular mass index (LVMI) = LVM/body surface area (BSA) × 100%. The average of more than three consecutive cardiac cycles was used as the experimental result.

### Immunohistochemistry

The rat heart tissues were fixed in 4% formaldehyde, paraffin-embedded, and sectioned (5-μm). Paraffin-embedded sections were heated at 60 °C, dewaxed, hydrated in 3% H_2_O_2_ for antigen retrieval, and then incubated with 5% bovine serum albumin at 37 °C for 0.5 h. The sections were probed with primary antibody to Ki-67 (1:300, ab92742, Abcam, Cambridge, UK) overnight at 4 °C and then incubated with goat anti-rabbit secondary antibody (1:1000, ab205718, Abcam) for 1 h at 37 °C. Streptavidin-peroxidase solution (Solarbio, Beijing, China) was added and incubated for 0.5 h at 37 °C before diaminobenzidine (DAB) staining. The sections were soaked in hematoxylin for 5 min and viewed under a microscope (Zeiss AxioX-4, Carl Zeiss, Oberkochen, Germany). The Ki-67 activity was analyzed using Image-Proplus 6.0 (Imag J-FIJI-Trmk, Japan).

### RT-qPCR

RNA was extracted from tissue homogenates using the illustra RNAspin Mini RNA Isolation Kit (GE healthcare, Chicago IL, USA), and cDNA was produced using the High-Capacity cDNA Reverse Transcription Kit (Thermo Fisher Scientific Inc., Waltham, MA, USA). The transcriptional expression of genes was examined by Maxima SYBR Green/ROX qPCR Master Mix (2×) (Thermo Fisher Scientific) on a StepOnePlus™ Real-Time PCR system (Applied Biosystems, Foster City, CA, USA). The RT-qPCR primers are listed in Table 1. The mRNA expression was normalized to the expression of glyceraldehyde-3-phosphate dehydrogenase (GAPDH) and measured using the 2^−ΔΔCt^ cycle threshold method.

**Table 1:** The primer sequence

Gene	Sequence
EZH2	Forward: 5ʹ-AGTTCGTGCCCTTGTGTGAT-3’
	Reverse: 5ʹ-GAGGAGTTGTGTTTTCCCACT-3’
HMGA2	Forward: 5ʹ-CGAAAGGTGTTGGGCGGA-3’
	Reverse: 5’-GTTCTTGCTGCCTTTGGGTC-3’
Collagen I	Forward: 5’-GGAGAGAGCATGACCGATGG-3’
	Reverse: 5’-GGTGGGAGGGAACCAGATTG-3’
Collagen III	Forward: 5’-TTCCTGGGAGAAATGGCGAC-3’
	Reverse: 5’-ACCAGCTGGGCCTTTGATAC-3’
TGF-β1	Forward: 5’-AGGAGACGGAATACAGGGCT-3’
	Reverse: 5’-CCACGTAGTAGACGATGGGC-3’
CTGF	Forward: 5’-ACTGTTGGCGAACAAATGGC-3’
	Reverse: 5’-CTGCCTCCCAAACCAGTCAT-3’
GAPDH	Forward: 5’-GCATCTTCTTGTGCAGTGCC-3’
	Reverse: 5’-GATGGTGATGGGTTTCCCGT-3’

EZH2, enhancer of zeste 2 polycomb repressive complex 2 subunit; HMGA2, high mobility group AT-hook 2; TGF-β1, transforming growth factor beta 1; CTGF, CCN family member 2; GAPDH, glyceraldehyde-3-phosphate dehydrogenase

### Masson’s staining

Paraffin-embedded sections of rat hearts were dewaxed, stained with hematoxylin (Sigma-Aldrich Chemical Company, St Louis, MO, USA) for 2 min and with Ponceau S, and rinsed quickly with 0.5% glacial acetic acid. The sections were subjected to 1% aqueous aluminum phosphate (from dark red to bright red to pink staining) and aniline blue (Sigma-Aldrich) staining procedures, xylene dehydration, and sealing. The area of positive staining of collagen fibers was observed under a microscope (Zeiss AxioX-4, Zeiss) and measured using the Image-Proplus 6.0 (ImagJ-FIJI-Trmk, Japan). Collagen volume fraction (CVF) = collagen area/total field of view area. Cardiomyocytes were stained in red, whereas collagen fibers were in blue stripes or intercellular homogeneous structures.

### TUNEL assay

Paraffin-embedded sections of rat hearts were dewaxed, rehydrated, heated in citrate buffer at 350 W for 10 min, reacted with 50 μL TUNEL solution (Solarbio), 50 μL transformer-peroxidase, and DAB for 0.5 h. The sections were placed in hematoxylin, treated with 95% ethanol I–II, with anhydrous ethanol I–II, xylene I–II and sealed with neutral resin. Apoptotic rate (TUNEL-positive cells/total cells × 100%) was analyzed under a light microscope (Zeiss AxioX-4, Zeiss).

### Western blot

The protease inhibitor phenylmethanesulfonyl fluoride was mixed with cell lysis buffer (Thermo Scientific) at 1:100 for the extraction the proteins from the cells. The total protein concentration was measured with a bicinchoninic acid assay kit (Beyotime, Shanghai, China). The proteins were separated by electrophoresis on a 12% sodium dodecyl sulfate polyacrylamide gel after mixing with 5 × loading buffer at 4:1 and transferred onto PVDF membranes (Millipore Corp, Billerica, MA, USA). The membranes were sealed with 5% bovine serum albumin and subsequently incubated with the primary antibodies to EZH2 (39875, 1:1000, Active Motif., Carlsbad, CA, USA), HMGA2 (NBP2-43640, 1:1500, Novus Biological Inc., Littleton, CO, USA), H3K27me3 (ab6002, 1:1000, Abcam), p-PI3K (#17366, 1:1300, Cell Signaling Technologies, Beverly, MA, USA), PI3K (sc-390916, 1:1500, Santa Cruz Biotechnology Inc., Santa Cruz, CA, USA), p-AKT (NB100-56749, 1:1000, Novus Biological), AKT (sc-5298, 1:2000, Santa Cruz Biotechnology), E-cadherin (ab1416, 1:1000, Abcam), α-SMA (A2547, 1:1500, Millipore), FSP1 (370004, 1:2000, BioLegend, San Diego, CA, USA) and GAPDH (ab8245, 1:3000, Abcam). Horseradish peroxidase-labeled IgG secondary antibody (ab205719, 1:5000, Abcam) was then added dropwise to the membrane. The membrane was immersed in ECL (Pierce, Rockford, IL, USA), and Quantity One software (Bio-Rad Laboratories, Hercules, CA, USA) was utilized to analyze the protein blot images.

### MSC extraction and identification

The experimental animals were specific pathogen free-grade Sprague–Dawley male rats (Charles River), which were euthanized by injection of 1% sodium pentobarbital (120 mg/kg) intraperitoneally. After removal and sterilization with 75% alcohol, the femur and tibia were placed on an ultra-clean table. Muscle and connective tissues were removed, and the bone marrow cavity was repeatedly flushed with low-glucose Dulbecco's modified Eagle's medium (Sigma, Saint Louis, MO, USA). The liquid was centrifuged, and the precipitates were suspended and cultured for 24 h (medium was refreshed every 2–3 days). When grown to a logarithmic phase, MSCs were dissociated with 0.25% trypsin (Gibco, Carlsbad, CA, USA), centrifuged, and resuspended in the growth medium (Cyagen, Suzhou, Jiangsu, China).

MSCs at passage 4 were seeded at 200 cells/mL into 6-well plates. The osteogenic and adipogenic induction solution (Cyagen) were added to 60–70% confluent MSCs, and MSCs without induction solution were used as controls. MSCs were induced for two weeks and fixed with 4% paraformaldehyde, followed by alizarin red staining and oil red O staining (Beyotime). For alizarin red staining, the samples were covered with alizarin red staining solution and incubated for at least 60 min at 37 °C in the dark. The stained slides were rinsed slowly with double-distilled water for 3–5 min, followed by observation under a microscope (Zeiss AxioX-4, Zeiss). In the process of oil red O staining, 1 mL of staining solution was added for a 10–20 min culture. After the removal of the oil red O staining working solution, the slides were rinsed with the appropriate amount of staining washing solution for 30 s. Photographs were taken under a microscope (Zeiss AxioX-4, Zeiss).

Surface antigens of MSCs at passage 4 were detected by flow cytometry. MSCs were dissociated with 1 mL 0.25% trypsin containing ethylenediaminetetraacetic acid (EDTA), centrifuged at 151 × *g*, and resuspended in phosphate-buffered saline (PBS) containing 2% fetal bovine serum to prepare single cell suspensions. Monoclonal antibodies (20 μL each) against CD34 (MA1-10,205, 1:200, Invitrogen Inc., Carlsbad, CA, USA), CD29 (303002, 1:500, BioLegend) and CD44 (ab6124, 1:1000, Abcam) were used to stain 100 μL cell suspension. The cell suspension was centrifuged at 151 × g, fixed in 1% paraformaldehyde for 0.5 h, and loaded onto a FACSCanto (BD Bioscience, San Jose, CA, USA) flow cytometer. Analysis was performed by FlowJo 8.7.1 software (TreeStar, Ashland, OR, USA).

### MSC-EXO isolation and identification

The supernatant of MSCs cultured for 48 h was subjected to a series of centrifugation at 300 *g* for 5 min, at 1200 *g* for 20 min, at 10,000 *g* for 30 min, and at 100,000 g (Sorvall WX Ultra, Thermo Fisher). The precipitate was resuspended in PBS and centrifuged again at 100,000 *g* to obtain the EXO precipitate.

The extracted EXO were resuspended using PBS, fixed with 2% paraformaldehyde and loaded on carbon-coated copper grids. The grids were placed on 2% gelatin at 37 °C for 20 min and washed with 0.15 M glycine in PBS. Then, the morphology of MSC-EXO was viewed under a transmission electron microscope (CM120, Philips Research, Eindhoven, The Netherlands). The marker proteins (CD63, CD81 and CD9) were detected by protein blot analysis, and the primary antibodies used for the analysis were as follows: CD63 (ab8219, 1:2000, Abcam), CD81 (sc-23962, 1:1500, Santa Cruz Biotechnology) and CD9 (AHS0902, 1:1000, Invitrogen). For control, MSC lysate free of EXO was used, and the supernatant obtained in ultracentrifugation was the lysate. The analytical methods were consistent with western blot methods.

### Bioinformatics analysis

The construction of the associated gene cluster and protein–protein interactions (PPI) of EZH2 were performed through the STRING website (https://string-db.org/) with confidence = 0.90. The enriched regions of EZH2 and H3K27me3 on the HMGA2 promoter were obtained from ChIPBase v3.0 (http://rna.sysu.edu.cn/chipbase3). KEGG analysis of genes was performed on R scripts via the ClusterProfiler package (Bioconductor, Seattle, WA, USA) and KEGG pathway data were downloaded from the KEGG database (https://www.kegg.jp/kegg/rest/keggapi.html).

### Chromatin immunoprecipitation (ChIP)

ChIP was performed in H9C2 rat cardiomyocytes (American Type Culture Collection, Manassas, VA, USA). H9C2 cells (5 × 10^6^) were placed in 15-cm culture dishes, cross-linked with 1% formaldehyde, and quenched with glycine. Nuclease was added to lyse the nuclei for 20 min, and the reaction was terminated with 0.5 mol/L EDTA, followed by ultrasonic disruption of the nuclear membrane. After centrifugation, the supernatant containing chromatin was collected, and the chromatin solution was incubated with antibody to EZH2 (1:100, ab191250, Abcam) or the isotype control IgG (1:500, ab172730, Abcam) overnight at 4 °C. The beads were then incubated with ChIP level protein G magnetic beads (Thermo Fisher Scientific) for 3 h at 4 °C and washed. The crosslinking was reversed by incubation at 65 °C for 2 h. The de-crosslinked DNA was purified and subjected to PCR analysis.

### Statistical analysis

SPSS 22.0 software (IBM Corp. Armonk, NY, USA) was utilized for analyses. The data were expressed as mean ± SD of at least three experiments for every assay. The unpaired *t* test was used for two-group comparisons. One- or two-way ANOVA was utilized for multiple-group comparisons, and Tukey’s post hoc test. Predictors were kept if they were significant at a *p* value of 0.05 or smaller.

## Results

### MI contributes to cardiac function decline in rats

**Fig. 1:**
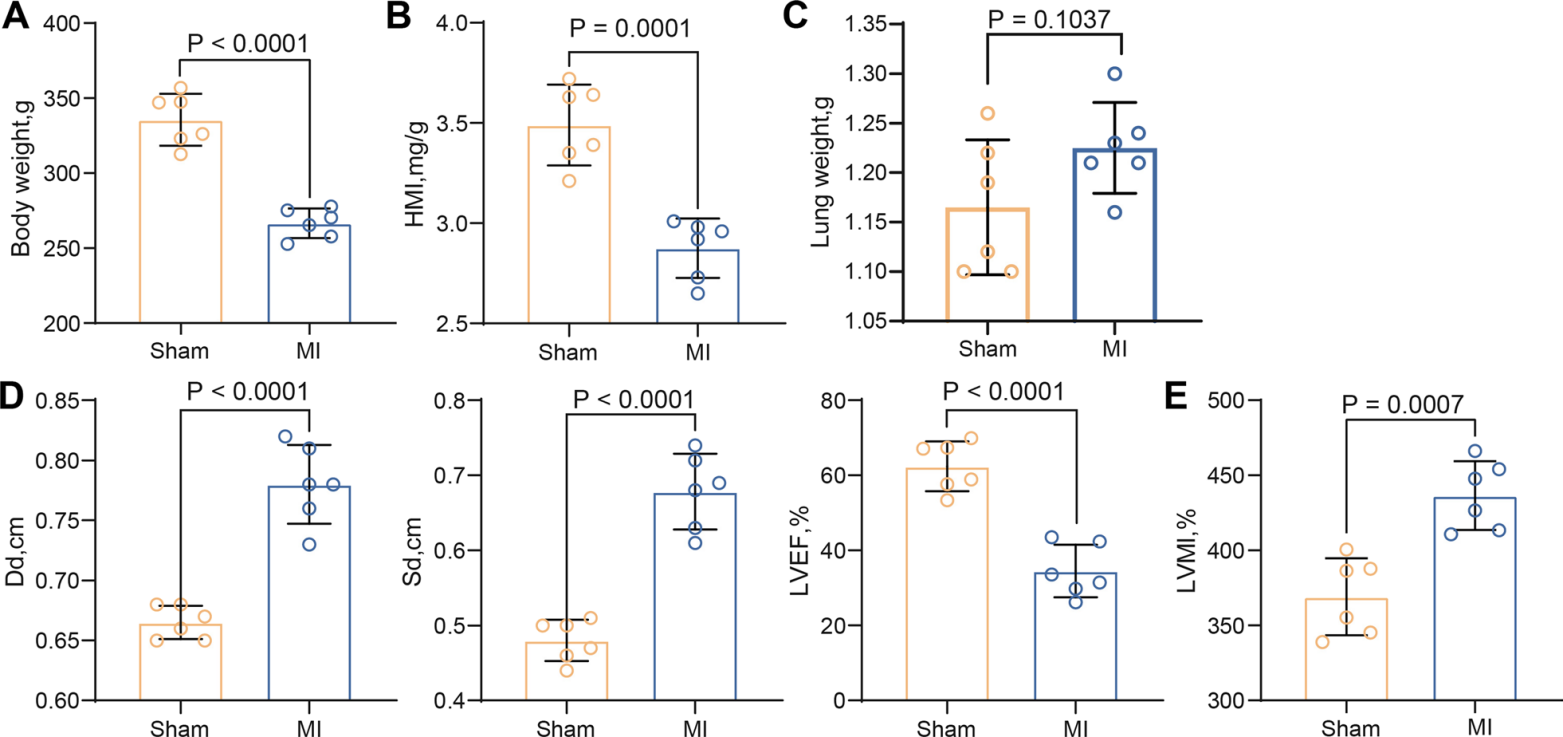
MI leads to cardiac dysfunction in rats. **A** Weight change in rats after MI modeling. **B** Changes in HMI in rats after MI modeling. **C** Detection of lung tissue weight in rats. **D** Measurement of Dd, Sd, and LVEF by echocardiography at 2 weeks after LAD in rats. **E** Measurement of LVMI by echocardiography in rats. All data are expressed as mean ± SD (n = 6/group, *p < 0.05 vs. sham group determined by unpaired t test)

We established an MI model in Sprague–Dawley rats and examined the body, heart and lung weights of the rats. A statistically significant reduction in body weight was shown in the MI rats (Fig. 1A). Heart mass index (HMI) was significantly lower in all MI rats compared to the sham-operated rats (Fig. 1B), and no significant difference was found in the lung weight (Fig. 1C). These findings confirmed significant myocardial damage in rodents underwent LAD ligation. Cardiac function was assessed by echocardiography, and all MI rats exhibited significantly higher Dd and Sd, whereas lower LVEF 2 weeks after LAD surgery compared to sham-operated rats (Fig. 1D). Meanwhile, LVMI was significantly higher in MI rats (Fig. 1E). Therefore, these data suggest that the MI model was successfully constructed.

### MI leads to myocardial fibrosis in rats

**Fig. 2:**
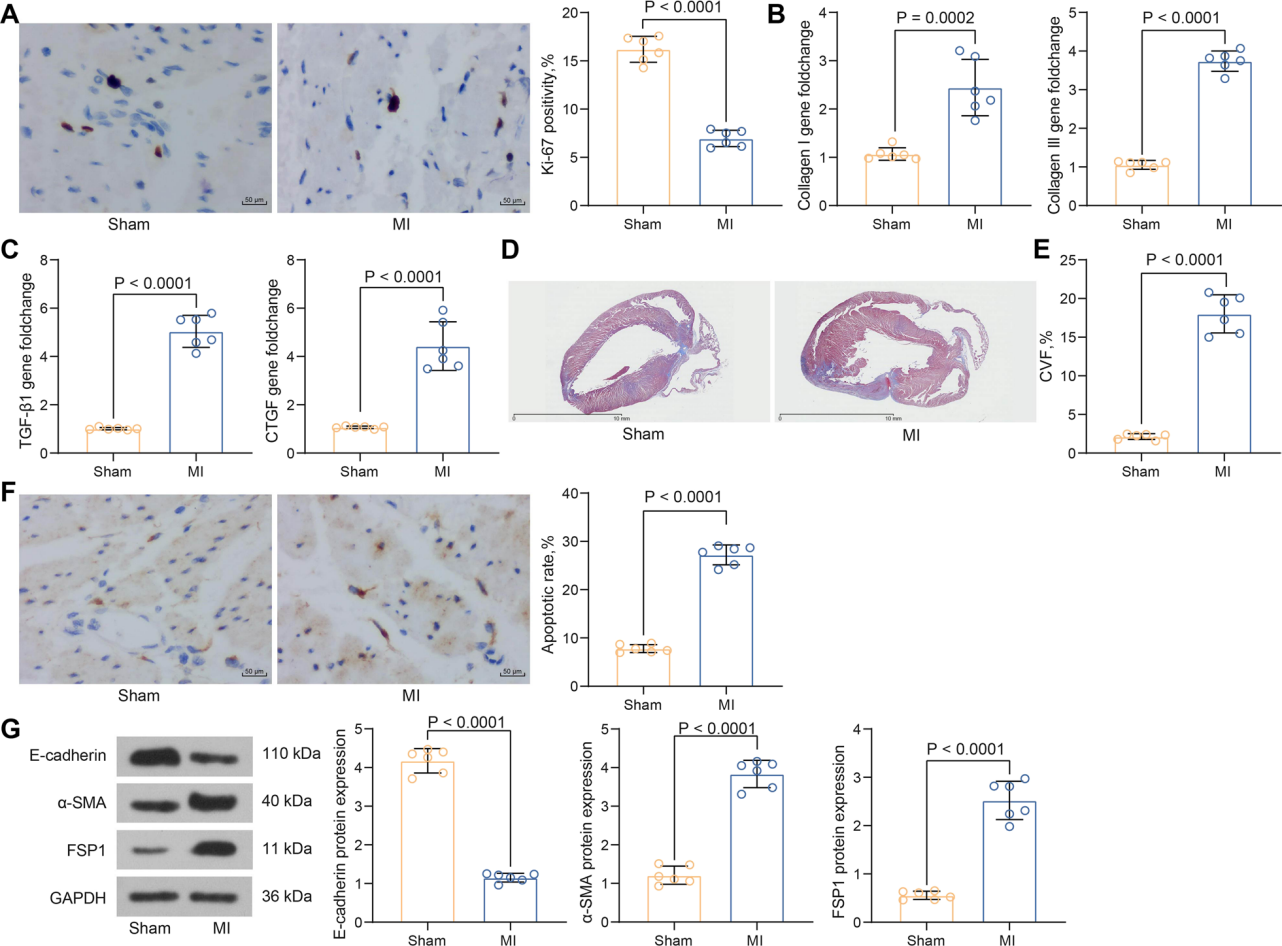
MI leads to myocardial fibrosis in rats. **A** Intensity of ki-67 staining in rat myocardial tissues assessed by immunohistochemistry. **B** Collagen I and collagen III expression in rat myocardial tissues assessed by PCR analysis. **C** Detection of TGF-β1 and CTGF expression in rat myocardial tissues assessed by RT-qPCR. **D** Fibrosis of rat myocardial tissue assessed using Masson’s staining. **E** CVF in rat myocardium. **F** Apoptosis in rat myocardial tissues assessed using TUNEL. **G** E-cadherin, α-SMA, FSP1 protein expression in rat myocardial tissues assessed using western blot (Additional file 1: Fig. S1). All data are expressed as mean ± SD (n = 6/group, *p < 0.05 vs. sham group determined by unpaired t test)

Cardiomyocyte viability was detected in tissues by assessing the intensity of ki-67 staining. The results of immunohistochemistry showed a significant decrease in the viability of cardiomyocytes (i.e., working cells) in the atria and ventricles in MI rats (Fig. 2A). RT-qPCR was then used to determine the expression of collagen I and collagen III in myocardial tissues. They were both significantly upregulated in MI rats (Fig. 2B). The RT-qPCR results also exhibited that the levels of TGF-β1 and CTGF were elevated in MI rats as well (Fig. 2C). Only minimal blue-stained fibrous tissue was observed in the sham-operated rats using Masson’s staining, and cells in the myocardial tissues of MI rats were disorganized with a significant augment in myocardial fibers and enlarged blue-stained areas (Fig. 2D). In addition, the CVF of myocardial tissues in rats was calculated, and the results exhibited that the CVF in the MI group was much higher than that in the sham group (Fig. 2E). TUNEL assay showed that apoptotic cells were brownish-black or brownish-yellow with cohesive nuclei, and the apoptotic rate of working cells was boosted in MI rats (Fig. 2F). E-cadherin, α-SMA and FSP-1 are the crucial biomarkers of EMT. E-cadherin protein expression was decreased and α-SMA and FSP1 protein expression was increased in MI rats, as revealed by western blot (Fig. 2G).

### Characterization of MSC and MES-EXO

**Fig. 3:**
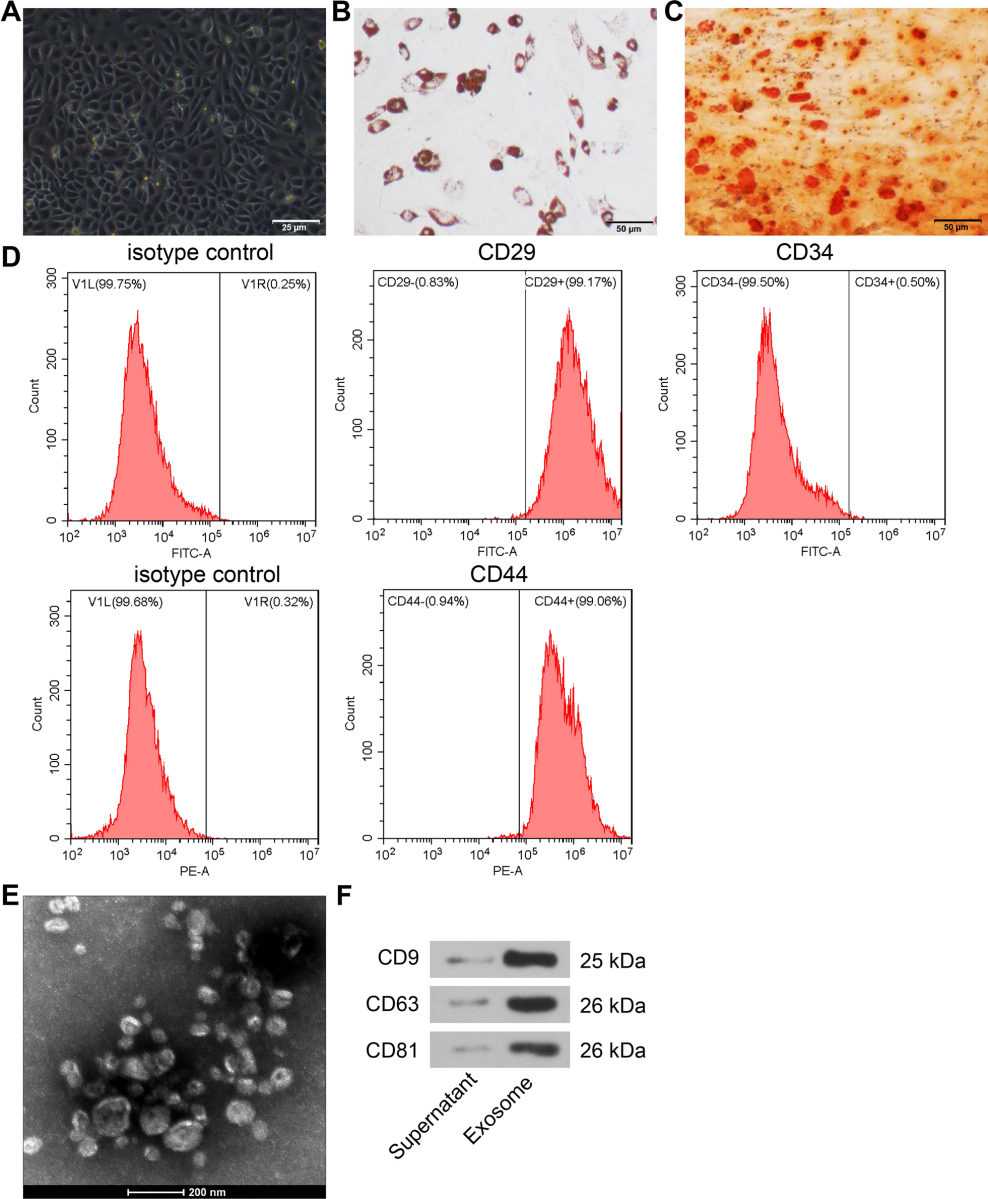
Observation of MSC phenotype and identification of MSC-EXO. **A** Observation of MSC morphology under light microscope. **B** Representative results of adipocytes using oil red O staining. **C** Representative results of osteoblast using alizarin red staining. **D** MSC phenotype detected by flow cytometry. **E** Electron microscopic observation of MSC-EXO. **F** CD9, CD63 and CD81 protein blots in MSC-EXO and supernatant (MSC cell lysate excluding EXO) by western blot (Additional file 2: Fig. S2)

Under the microscope, bone marrow-derived MSC were spindle-shaped and round and attached to the wells in a vortex or radial pattern (Fig. 3A). After adipogenic differentiation, MSCs showed rounded red lipid droplets of various sizes (Fig. 3B). After osteogenesis induction, the cells expressing calcified nodules were stained in red, and the calcified nodules were unevenly distributed with overlapping cells (Fig. 3C). Flow cytometry showed expression of MSC markers CD29 and CD44 (> 95%), but not of CD34 (< 95%), the hematopoietic stem cell surface antigen (Fig. 3D). These results indicate that the bone marrow-derived MSCs are of high purity and meet the standards of the International Society for Cell Therapy.

BMSC-EXO were observed by transmission electron microscopy as ellipsoidal vesicles with clear peripheral membrane structure and variable size (40–100 nm in diameter) (Fig. 3E). The extracted products expressed CD9, CD63 and CD81, as Western blot analysis suggested (Fig. 3F).

### MSC-EXO alleviate cardiac dysfunction via EZH2 in rats with MI

**Fig. 4:**
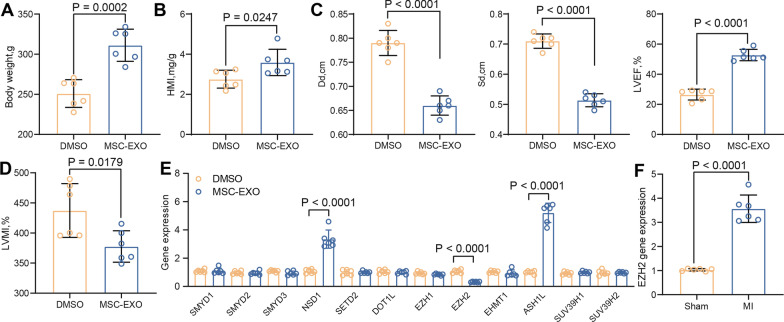
MSC-EXO enhance cardiac function in MI rats. **A**, Weight change in rats after MSC-EXO treatment. **B** Changes in HMI in rats after MSC-EXO treatment. **C**, Measurement of Dd, Sd, and LVEF by echocardiography at 2 weeks after LAD in rats. **D** Measurement of LVMI by echocardiography in rats. **E** mRNA expression of epigenetic modifiers in rat myocardial tissues examined using RT-qPCR. **F** PCR detection of EZH2 expression in myocardial tissues of MI rats. All data are expressed as mean ± SD (n = 6/group, *p < 0.05 vs. Sham or DMSO group determined by unpaired t test or two-way ANOVA)

MI rats were treated with MSC-EXO, and a gradual improvement in appetite and partial body gain were observed (Fig. 4A). Significant HMI elevation in rats was found upon MSC-EXO treatment at 2 weeks after surgery (Fig. 4B). Meanwhile, MSC-EXO treatment caused a significant decrease in Dd and Sd, whereas a remarkable increase in LVEF (Fig. 4C). Consistently, a significant decline in LVMI was observed in MI rats (Fig. 4D). It indicates that the treatment of MSC-EXO partially restored the cardiac function in rats. To probe the molecular mechanisms during the treatment of MSC-EXO, we focused on epigenetic mechanisms and found that EZH2 was significantly inhibited after MSC-EXO treatment (Fig. 4E). EZH2 was detected using RT-qPCR to be significantly elevated in the myocardial tissues of MI rats (Fig. 4F), suggesting that MSC-EXO alleviate myocardial injury in rats through inhibition of EZH2.

### EZH2 inhibits HMGA2 expression via elevating H3K27me3 modification

**Fig. 5:**
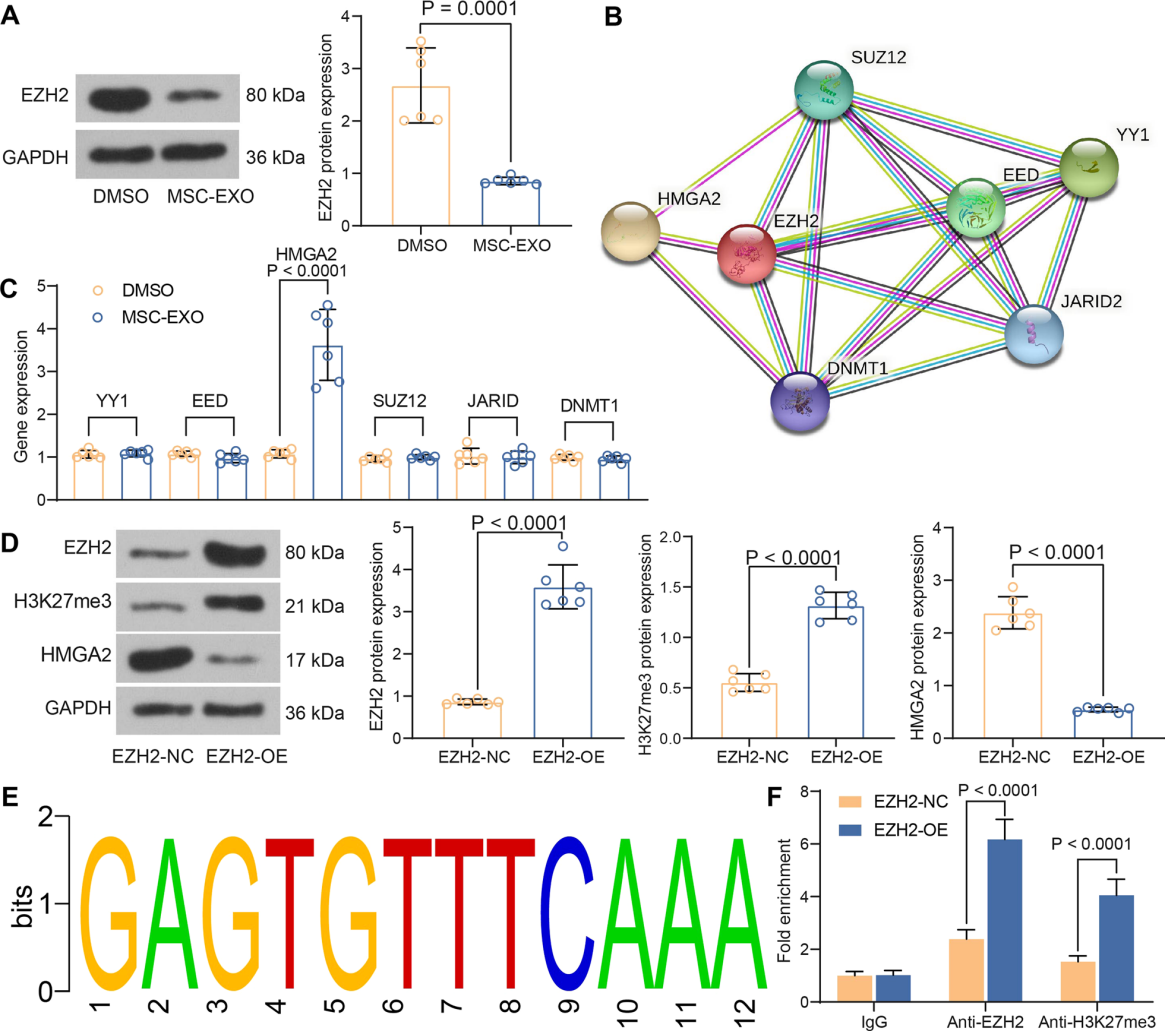
EZH2 and HMGA2 may involve in the cardio-protection provided by MSC-EXO in MI. **A** EZH2 protein expression in myocardial tissues in response to MSC-EXO examined using western blot (Additional file 3: Fig. S3). **B** Related gene clusters of EZH2 constructed by STRING. **C** expression of related genes in rat myocardial tissues in response to MSC-EXO measured using RT-qPCR. **D** Protein expression of EZH2, HMGA2 and H3K27me3 in rat myocardial tissues in response to EZH2-OE measured using western blot (Additional file 4: Fig. S4). **E** Analysis of the binding sites of EZH2 and H3K27me3 on the HMGA2 promoter by bioinformatics prediction. **F** The recruitment of EZH2 and H3K27me3 on the HMGA2 promoter verified using ChIP assay. All data are expressed as mean ± SD (n = 6/group, *p < 0.05 vs. DMSO, EZH2-NC or IgG group determined by unpaired t test or two-way ANOVA)

The protein expression of EZH2 was measured in MSC-EXO-treated rats, and it was found that EZH2 protein expression decreased after MSX-EXO treatment (Fig. 5A). Since EZH2 plays the role of histone demethylase in diseases to regulate other genes, a PPI network was constructed to detect the downstream genes of EZH2. Six genes were found to be downstream of EZH2 (Fig. 5B). The detection of these genes using RT-qPCR revealed that HMGA2 was significantly increased after EXO treatment relative to other five genes (Fig. 5C). Therefore, we selected HMGA2 as a downstream gene of EZH2.

Rats overexpressing EZH2 were constructed after treatment with MSC-EXO, and elevation of H3K27me3 and downregulation of HMGA2 were detected in these rats (Fig. 5D). Figure 5E exhibits the binding site between EZH2 and HMGA2 promoter region. The elevation of EZH2 was found to significantly promote the enrichment of EZH2 and H3K27me3 at the same binding site on the HMGA2 promoter in H9C2 cells by ChIP assay (Fig. 5F).

### EZH2/HMGA2 mediates cardiac function in MI rats via the PI3K/AKT pathway

**Fig. 6:**
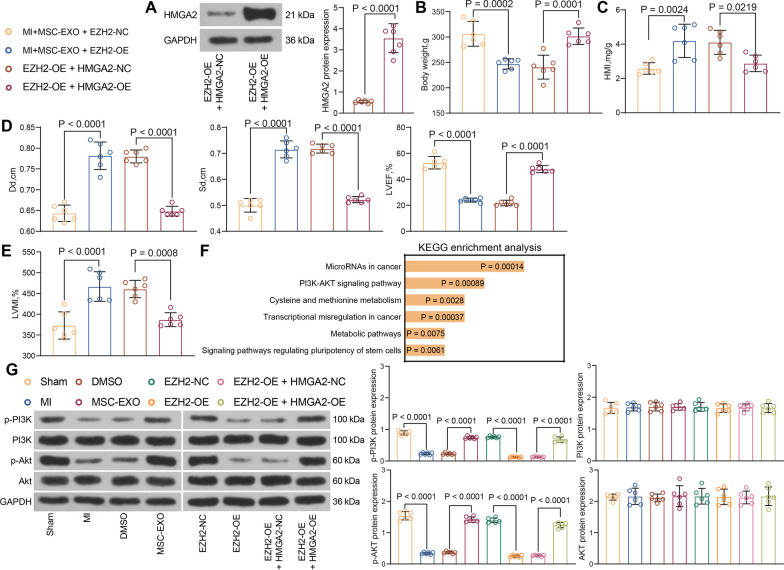
EZH2/HMGA2 regulates the PI3K/AKT signaling. MI rats were subjected to injection of EZH2-OE or EZH2-NC (MI + MSC-EXO + EZH2 NC/EZH2 OE) and EZH2-OE + HMGA2-OE or EZH2-OE + HMGA2-NC. **A** HMGA2 protein expression in myocardial tissues in response to EZH2-OE + HMGA2-OE examined using western blot (Additional file 5: Fig. S5). **B** Weight change in rats after EZH2-OE + HMGA2-OE treatment. **C** Changes in HMI in rats after EZH2-OE + HMGA2-OE treatment. **D** Measurement of Dd, Sd, and LVEF by echocardiography at 2 weeks after LAD in rats. **E** Measurement of LVMI by echocardiography in rats. **F** KEGG pathway analysis of gene enrichment pathways [29]. **G** Changes in PI3K/AKT pathway in rat myocardium examined using western blot (Additional file 6: Fig. S6). All data are expressed as mean ± SD (n = 6/group, *p < 0.05 vs. Sham, DMSO, MI + MSC-EXO + EZH2-NC or EZH2-OE + HMGA2-NC group determined by unpaired t test or one-way ANOVA)

EZH2 was upregulated alone or with HMGA2-OE in myocardial tissue of MI rats by administration of adeno-associated viruses containing vectors after MSC-EXO treatment. The delivery of EZH2-OE + HMGA2-OE elevated HMGA2 protein levels relative to EZH2-OE + HMGA2-NC (Fig. 6A). The upregulation of EZH2 reduced the body weight of rats, and HMGA2 induced partial recovery of body weight in rats (Fig. 6B). Also, HMI was reduced by EZH2 induction, and HMGA2 reversed the effect of EZH2 to increase HMI (Fig. 6C). Overexpression of EZH2 reduced Dd and Sd, while enhanced LVEF. By contrast, simultaneous HMGA2 overexpression elevated Dd and Sd, and decreased LVEF in MI rats (Fig. 6D). The trends for LVMI were consistent with HMI value (Fig. 6E). Evaluation of the downstream pathways of EZH2 and its associated gene cluster revealed that both were enriched in the PI3K/AKT pathway (Fig. 6F). The PI3K/AKT pathway activity was detected in rat myocardial tissues. It was found that the extent of PI3K and AKT phosphorylation was decreased in MI rat myocardial tissues, which was restored after MSC-EXO treatment. By contrast, EZH2 overexpression blocked the pathway activation, while HMGA2 reversed the effect of EZH2 to induce the PI3K/AKT pathway again (Fig. 6G). It indicates that EZH2/HMGA2 mediates the activity of PI3K/AKT pathway, thereby modulating the cardiac function in MI rats.

### MSC-EXO inhibit myocardial fibrosis via the EZH2/HMGA2 axis in MI rats

**Fig. 7:**
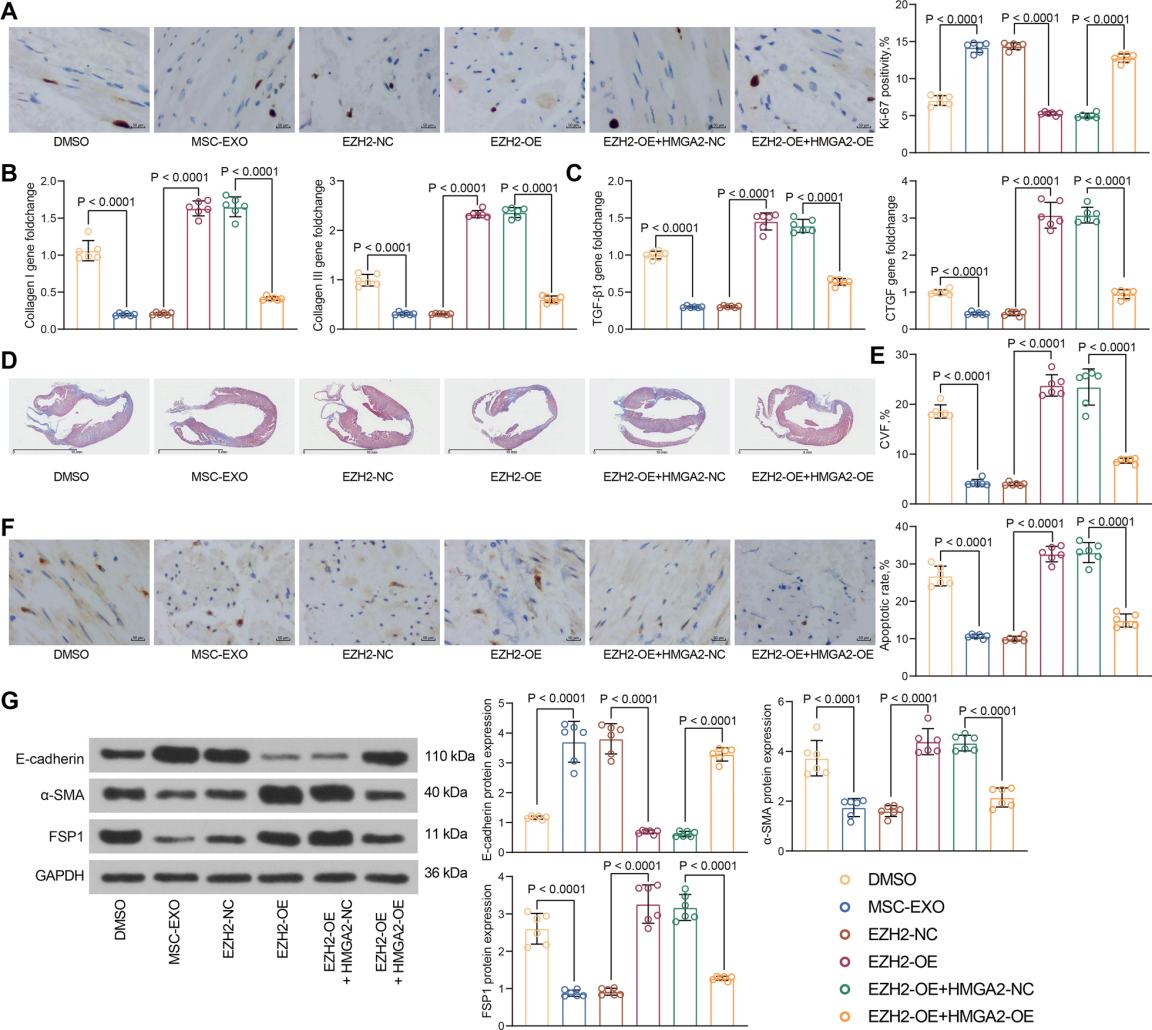
EZH2/HMGA2 regulates myocardial fibrosis in MI rats. **A** Intensity of ki-67 staining in myocardial tissues of rats by immunohistochemistry. **B** Collagen I and collagen III expression in myocardial tissues of rats examined using PCR analysis. **C** Detection of TGF-β1 and CTGF expression in myocardial tissues of rats by RT-qPCR. **D** Fibrosis of myocardial tissues of rats assessed using Masson’s staining. **E** CVF in rat myocardium. **F** Apoptosis in myocardial tissues of rats examined using TUNEL. **G** E-cadherin, α-SMA, FSP1 protein expression in myocardial tissues of rats examined using western blot (Additional file 7: Fig. S7). All data are expressed as mean ± SD (n = 6/group, *p < 0.05 vs. DMSO, MI + MSC-EXO + EZH2-NC or EZH2-OE + HMGA2-NC group determined by one-way ANOVA)

Ki-67 levels were increased by MSC-EXO treatment and significantly decreased by EZH2. However, HMGA2 reversed the effect of EZH2 to increase the proliferative activity of working cells (Fig. 7A). Meanwhile, MSC-EXO delivery decreased collagen I and collagen III expression, and EZH2 promoted collagen I and collagen III expression in rat myocardial tissues. HMGA2 overexpression, again, inhibited collagen I and collagen III expression (Fig. 7B). TGF-β1 and CTGF expression was reduced by MSC-EXO treatment, and the level of fibrosis-related genes was also significantly increased by EZH2. HMGA2 played an anti-fibrotic in rat myocardial tissues in the presence of EZH2 (Fig. 7C). It was found by Masson’s staining that MSC-EXO restored myocardial cell alignment in rats, while EZH2 exacerbated myocardial fibrosis. Consistently, HMGA2 reversed the effect of EZH2 and reduced myocardial fibers (Fig. 7D). The assessment of CVF and apoptotic cells revealed that both MSC-EXO and HMGA2 inhibited the collagen production and working cell apoptosis, while EZH2 enhanced collagen volume and cardiomyocyte apoptosis (Fig. 7E, F). Detection of EMT showed that EZH2 increased EMT, whereas MSC-EXO and HMGA2 inhibited EMT in myocardial tissues (Fig. 7G). Overall, the MSC-EXO delayed EMT and fibrosis in myocardial tissues of MI rats through inhibiting EZH2 and thus activating HMGA2.

## Discussion

Extracellular vesicles, comprising a population of cell-derived lipid vesicles including EXO, microvesicles and others, are potential carriers that could be used in wide-ranging applications towards regeneration [15]. EXO are a subset of extracellular vesicles with a size of approximately 30–100 nm [16]. For instance, Charles et al*.* have established that systemic MSC-EXO reduced myocardial infarct size in a porcine model [17]. In the present study, we established that EXO secreted from bone marrow-derived MSC exhibited a cardioprotective effect in MI by alleviating myocardial fibrosis and EMT. Second, the beneficial effects of MSC-EXO were partially mediated by suppressing EZH2. Finally, EZH2 blocks the PI3K/AKT signaling by repressing HMGA2 expression via H3K27me3 modification of its promoter.

The cardioprotective effects of MSC-EXO have been indicated via inhibition of cardiomyocyte senescence [18]. Here, we revealed the anti-fibrotic role of MSC-EXO in MI, as manifested by reduced TGF-β1 and CTGF expression in myocardial tissues of MI rats following MSC-EXO injection. Sygitowicz et al*.* have recently summarized that the TGF-β1-Smad signaling pathway, which induces the expression of genes encoding pro-fibrotic molecules, including CTGF and TGF-β1, plays a central role in mediating atrial fibrotic remodeling in atrial fibrillation [19]. Silencing of osteoglycin has been suggested to exert inhibitory effects on myocardial fibrosis and EMT in a mouse model of myocarditis via downregulating levels of TGF-β1, FSP-1, α-SMA and upregulating levels of E-cadherin [20]. Likewise, we provided evidence that MSC-EXO restored E-cadherin expression, while reducing FSP-1 and α-SMA expression in myocardial tissues of MI rats. These results established the mitigating effects of MSC-EXO on the EMT process of myocardial tissues, which was in line with a previous study regarding the role of MSC-EXO in viral myocarditis [21].

Pharmacologic inhibition of EZH2 or siRNA-mediated knockdown of EZH2 inhibited activation of renal interstitial fibroblasts in vitro and abrogated deposition of extracellular matrix proteins and expression of α-SMA in the obstructed kidney [22], indicating the pro-fibrotic effects of EZH2 in the kidney. Also, blockade of EZH2 with 3-DZNeP suppressed the EMT in mouse lung epithelial cell line through downregulation of TGF-β1 [23]. EZH2 has also been documented to be involved in the pathological process of hepatic fibrosis by regulating hepatic stellate cells [24]. In the present study, we also observed that EZH2 inhibition is at least partially responsible for the mitigating effects of MSC-EXO on myocardial fibrosis and EMT. Moreover, EZH2-mediated inhibition of KLF14 expression via H3K27me3 promoted hepatic stellate cell activation and liver fibrosis [25]. Consistently, using PPI network, we identified HMGA2 as a downstream gene of EZH2. Further ChIP assay verified the direct binding between HMGA2 promoter and EZH2. Under the condition of colorectal cancer, long noncoding RNA PiHL epigenetically activated HMGA2 transcription by relieving EZH2 on HMGA2, thus promoting PI3K/AKT phosphorylation [26].

In the present study, the KEGG enrichment analysis also revealed that the downstream genes of EZH2 were enriched in the PI3K/AKT pathway. For validation, we conducted western blot analysis, which demonstrated that the MSC-EXO treatment activated this pathway and EZH2 overexpression prevented the pathway from activation. Long-term exercise-derived circulating EXO have been suggested to protect the heart against myocardial ischemia/reperfusion injury by enhancing the extent of AKT phosphorylation [14]. HMGA2 contains five exons distributed over a genomic region of over 140 kb located at human chromosome 12q13-15 [27]. Cardiac-specific expression of HMGA2 in mice with an adeno-related virus 9 system ameliorated cardiac remodeling and improved cardiac function in response to pressure overload [28]. Our observation here showed that overexpression of HMGA2 downregulated the expression profile of collagen I, collagen III, TGF-β1, CTGF, FSP-1, α-SMA and promoted levels of E-cadherin in the presence of EZH2 overexpression.

This study also has several limitations. First, in addition to EZH2, whether other biomolecules that are mediated by MSC-EXO and involve in the cardioprotective effects of MSC-EXO requires further investigation. Second, further studies are required to see if dose, duration and timing of MSC-EXO treatment will further improve cardiac function post-MI. Third, the molecular mechanism between HMGA2 and the PI3K/AKT pathway need further elucidation.

## Conclusion

In conclusion, our study demonstrated that MSC-EXO is effective in improving heart function following MI. EZH2, which could be suppressed by MSC-EXO, impaired the PI3K/AKT pathway activation and HMGA2 expression via H3K27me3 modification in MI. Our study provides a novel candidate target to ameliorate fibrosis following MI.

## Supplementary Information

**Additional file 1:** Original, full-length gel and lot images of Fig 2G.

**Additional file 2:** Original, full-length gel and lot images of Fig 3F.

**Additional file 3:** Original, full-length gel and lot images of Fig 5A.

**Additional file 4:** Original, full-length gel and lot images of Fig 5D.

**Additional file 5:** Original, full-length gel and lot images of Fig 6A.

**Additional file 6:** Original, full-length gel and lot images of Fig 6G.

**Additional file 7:** Original, full-length gel and lot images of Fig 7G.

## Data Availability

All the data generated or analyzed during this study are included in this published article.

## Abbreviations

MSC-EXO: Mesenchymal stem cell-derived exosomes
MI: Myocardial infarction
EMT: Epithelial-mesenchymal transition
EZH2: Enhancer of zeste 2 polycomb repressive complex 2 subunit
HMGA2: High mobility group AT-hook 2
LAD: Left anterior descending
DMSO: Dimethylsulfoxide
LVESV: Left ventricular end-systolic diameter
LVEDV: Left ventricular end-diastolic diameter
LVEF: Left ventricular ejection fraction
BSA: Body surface area
DAB: Diaminobenzidine
GAPDH: Glyceraldehyde-3-phosphate dehydrogenase
CVF: Collagen volume fraction
TUNEL: Terminal deoxynucleotidyl transferase (TdT)-mediated 2′-Deoxyuridine 5′-Triphosphate (dUTP) nick end labeling
EDTA: Ethylenediaminetetraacetic acid
PBS: Phosphate-buffered saline
PPI: Protein–protein interactions
ChIP: Chromatin immunoprecipitation
