# Supplementary figures and images for: EZH2 mitigates the cardioprotective effects of mesenchymal stem cell-secreted exosomes against infarction via HMGA2-mediated PI3K/AKT signaling
Source: BMC Cardiovasc Disord. 2022 Mar 9;22:95. doi: 10.1186/s12872-022-02533-9 (PMC8908676; doi:10.1186/s12872-022-02533-9)

**Original, full-length gel and lot images of Fig 2G**

**
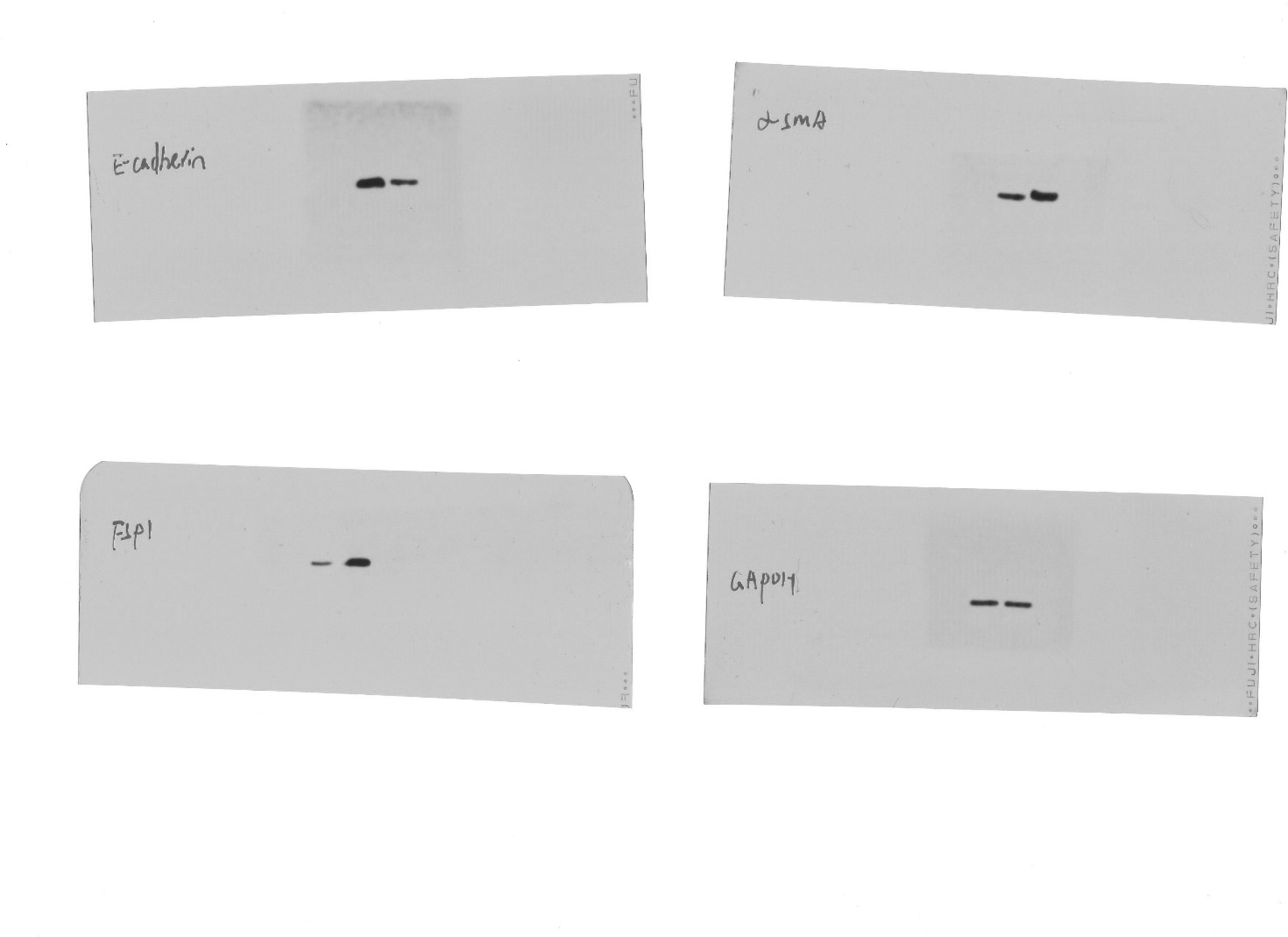
**

Supplement: Supplementary file 1 — Additional file 1: Original, full-length gel and lot images of Fig 2G. [file 12872_2022_2533_MOESM1_ESM.docx]

**Original, full-length gel and lot images of Fig 3F**

**
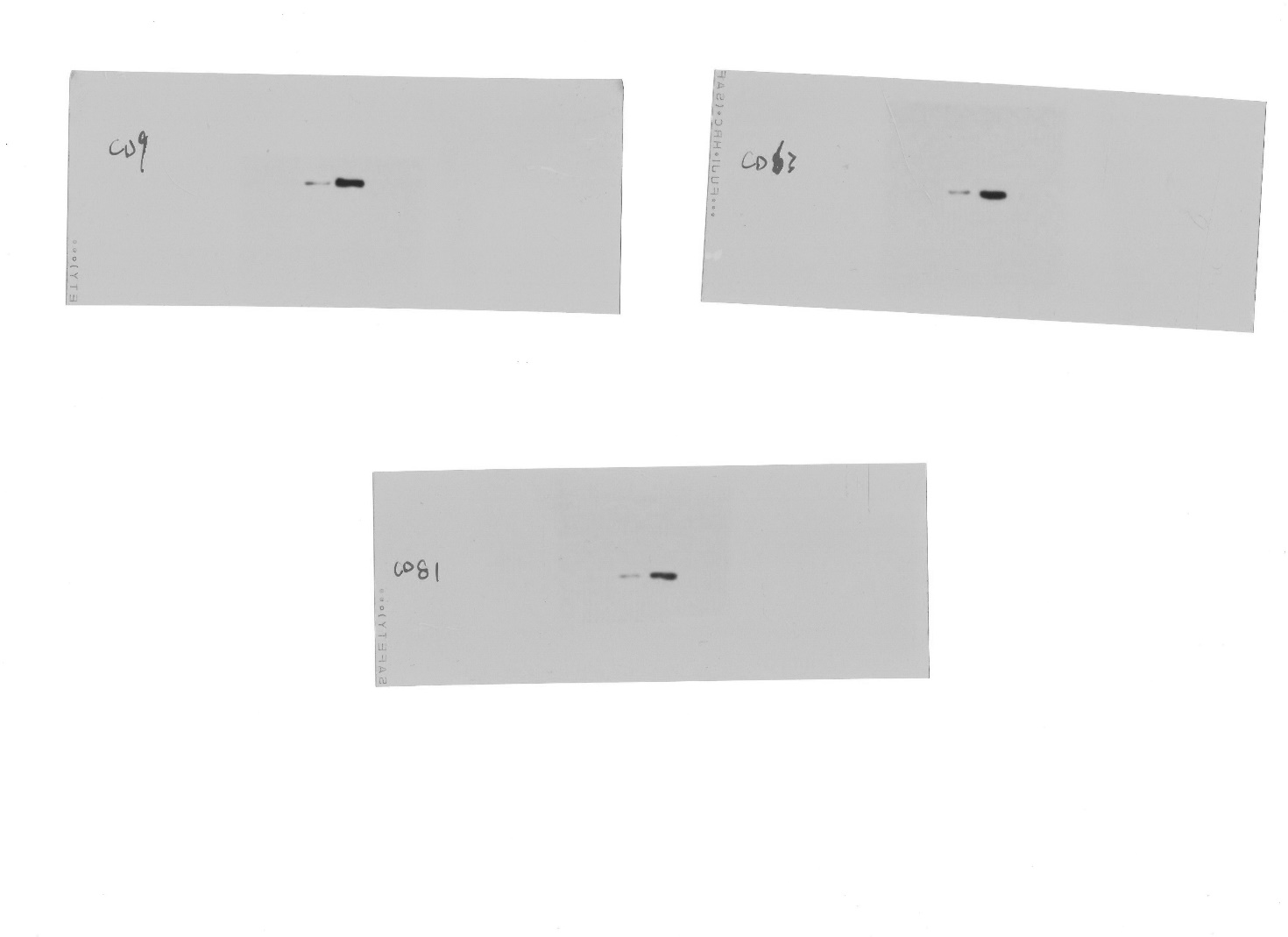
**

Supplement: Supplementary file 2 — Additional file 2: Original, full-length gel and lot images of Fig 3F. [file 12872_2022_2533_MOESM2_ESM.docx]

**Original, full-length gel and lot images of Fig 5A**

**
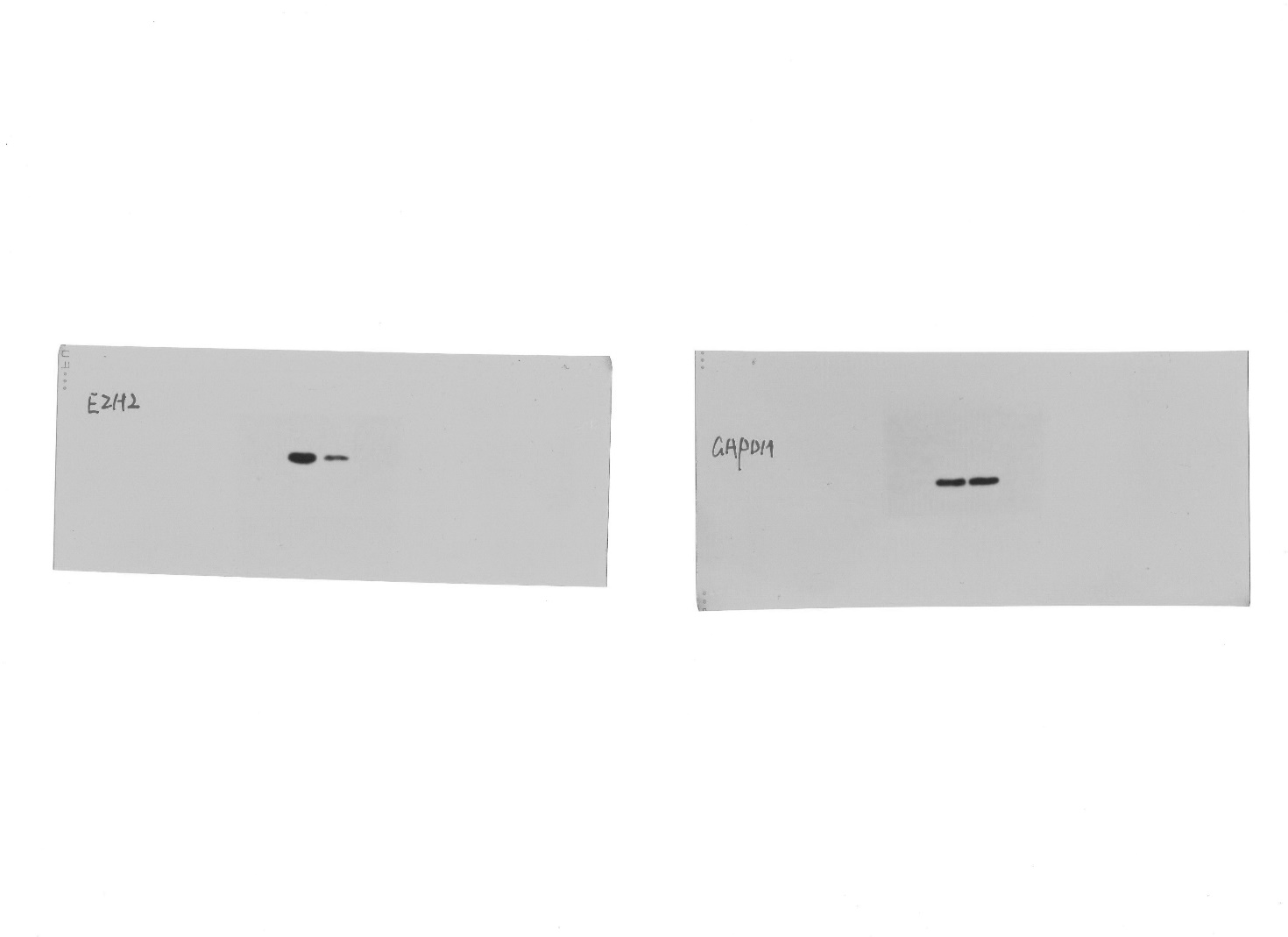
**

Supplement: Supplementary file 3 — Additional file 3: Original, full-length gel and lot images of Fig 5A. [file 12872_2022_2533_MOESM3_ESM.docx]

**Original, full-length gel and lot images of Fig 5D**

**
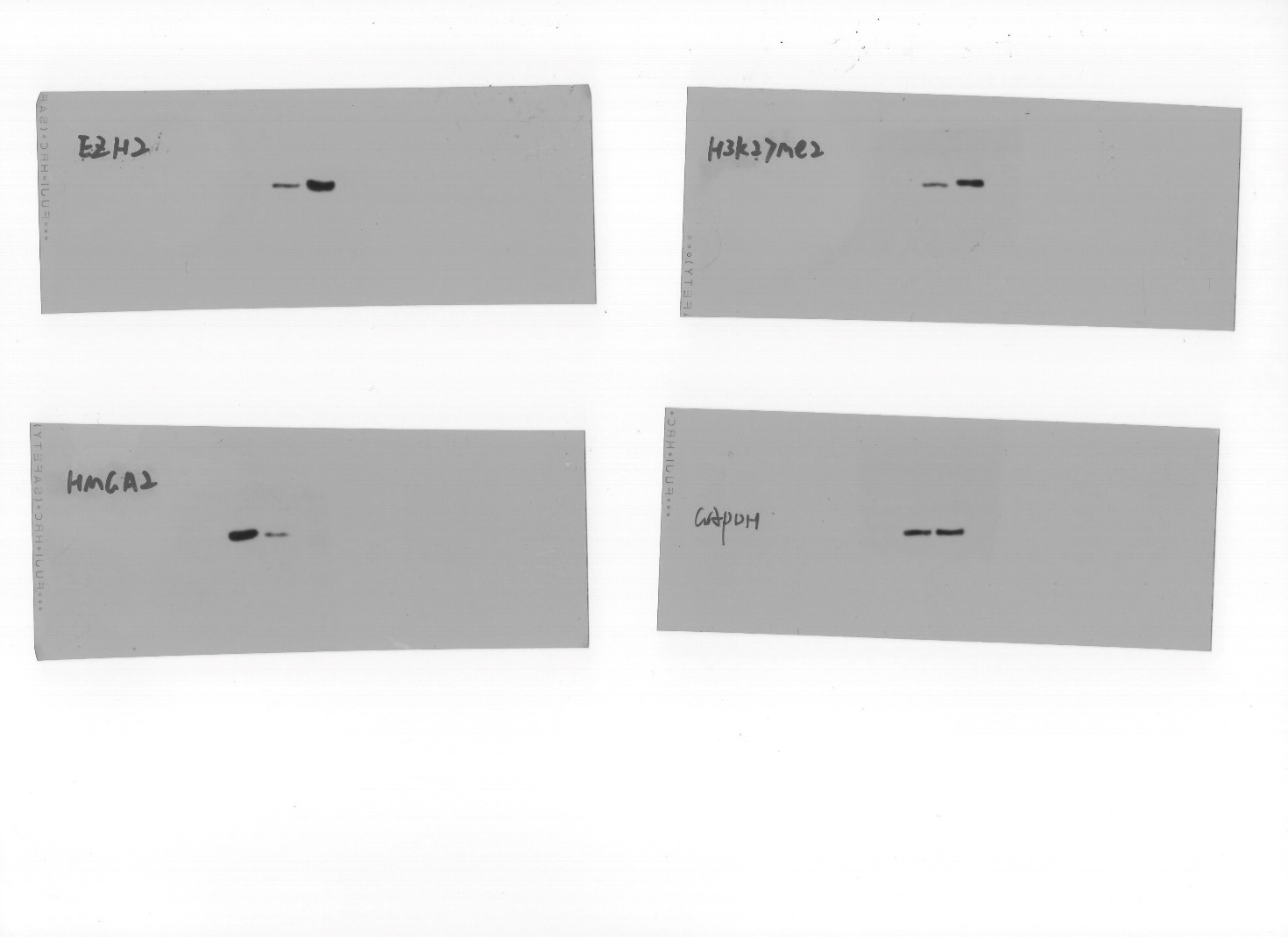
**

Supplement: Supplementary file 4 — Additional file 4: Original, full-length gel and lot images of Fig 5D. [file 12872_2022_2533_MOESM4_ESM.docx]

**Original, full-length gel and lot images of Fig 6A**

**
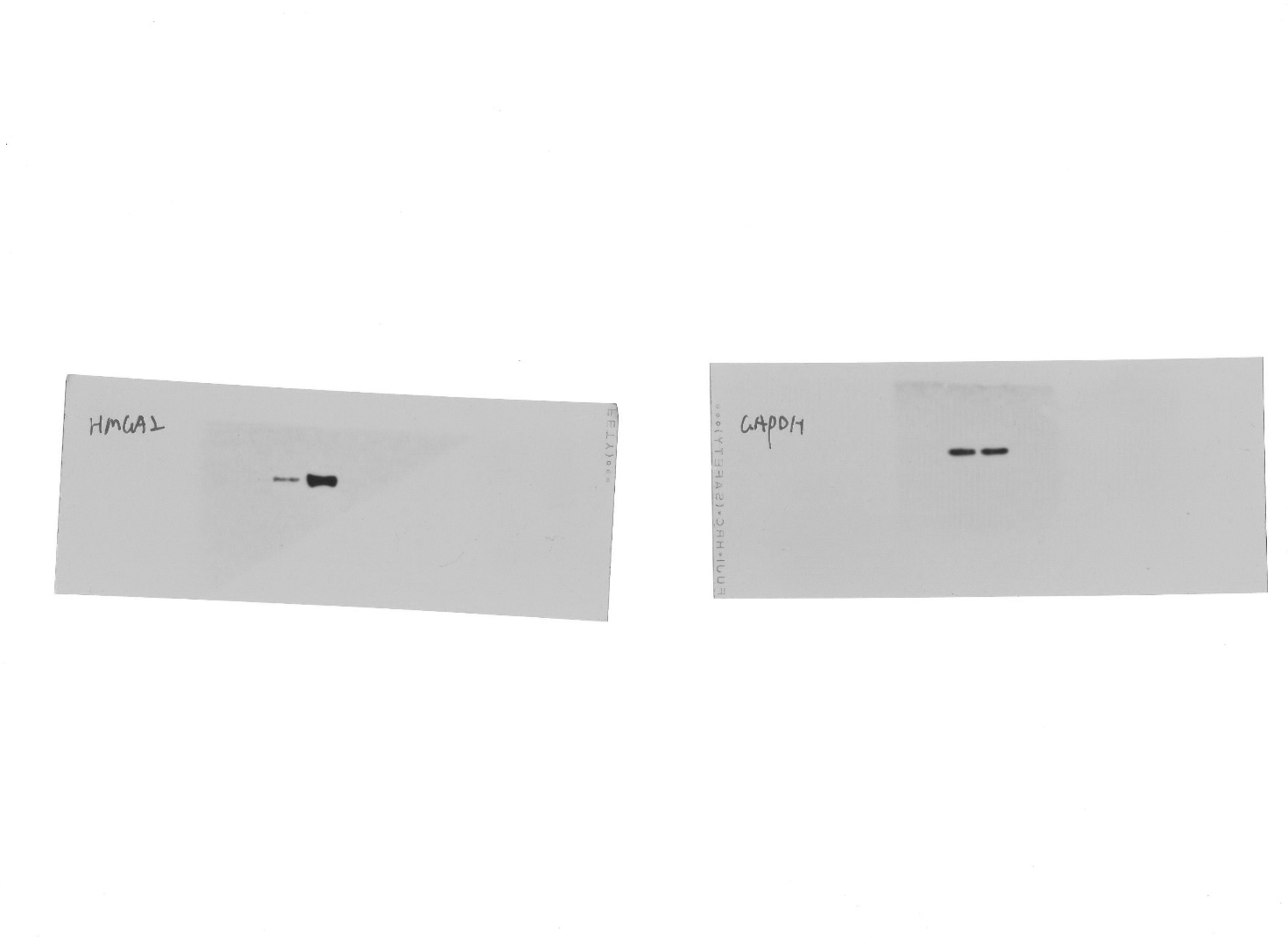
**

Supplement: Supplementary file 5 — Additional file 5: Original, full-length gel and lot images of Fig 6A. [file 12872_2022_2533_MOESM5_ESM.docx]

**Original, full-length gel and lot images of Fig 6G**

**
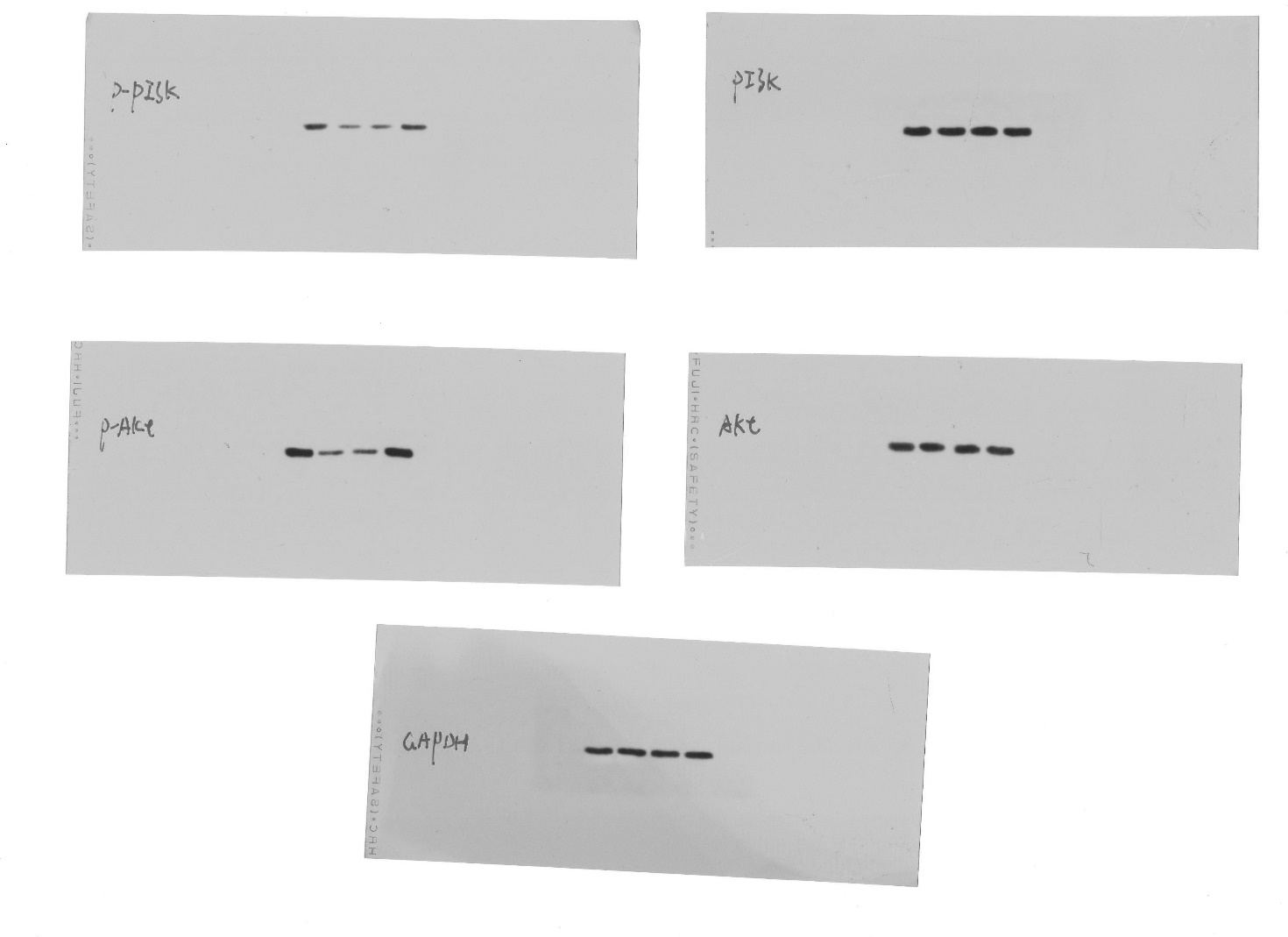
**

**
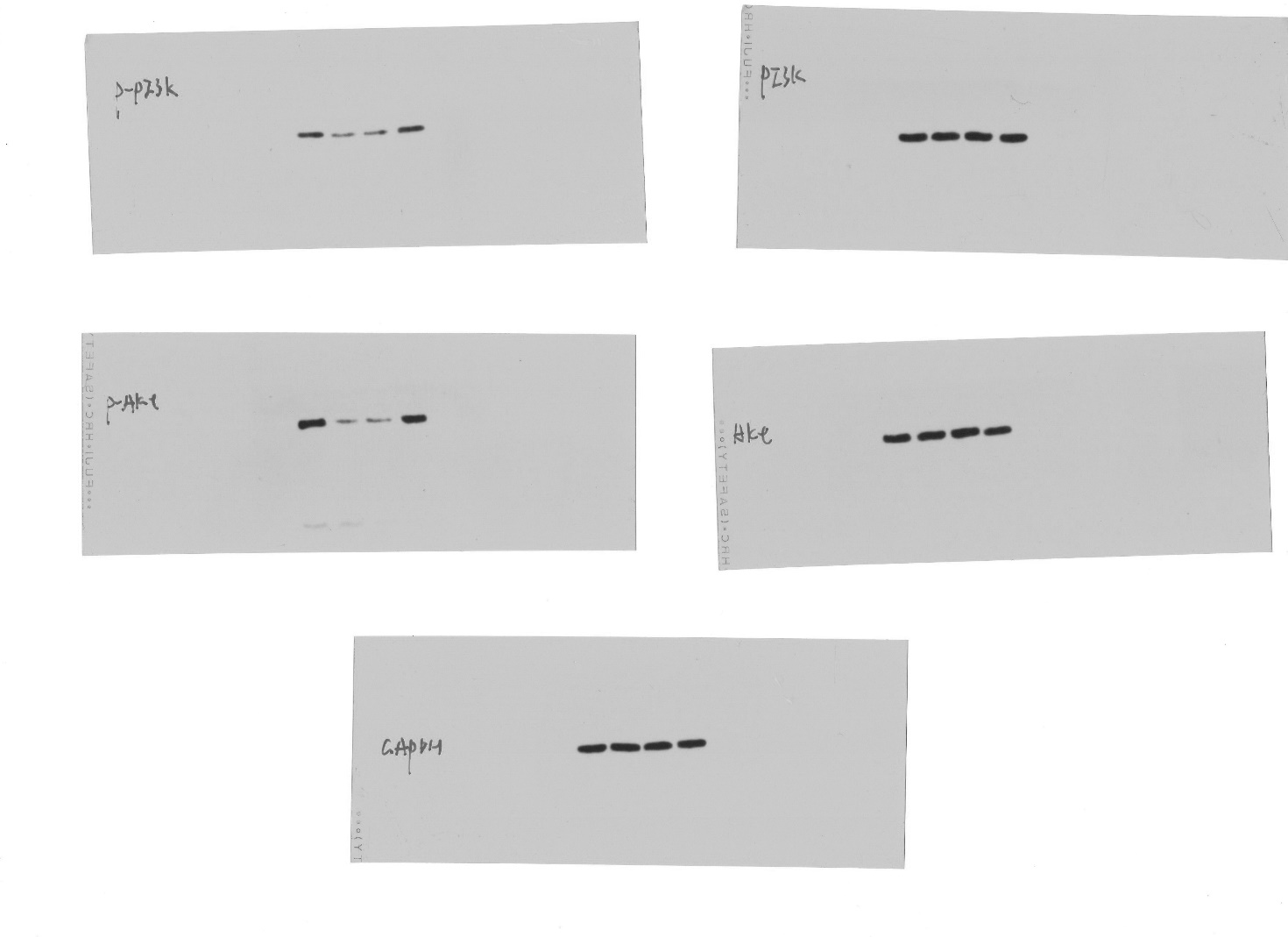
**

Supplement: Supplementary file 6 — Additional file 6: Original, full-length gel and lot images of Fig 6G. [file 12872_2022_2533_MOESM6_ESM.docx]

**Original, full-length gel and lot images of Fig 7G**

**
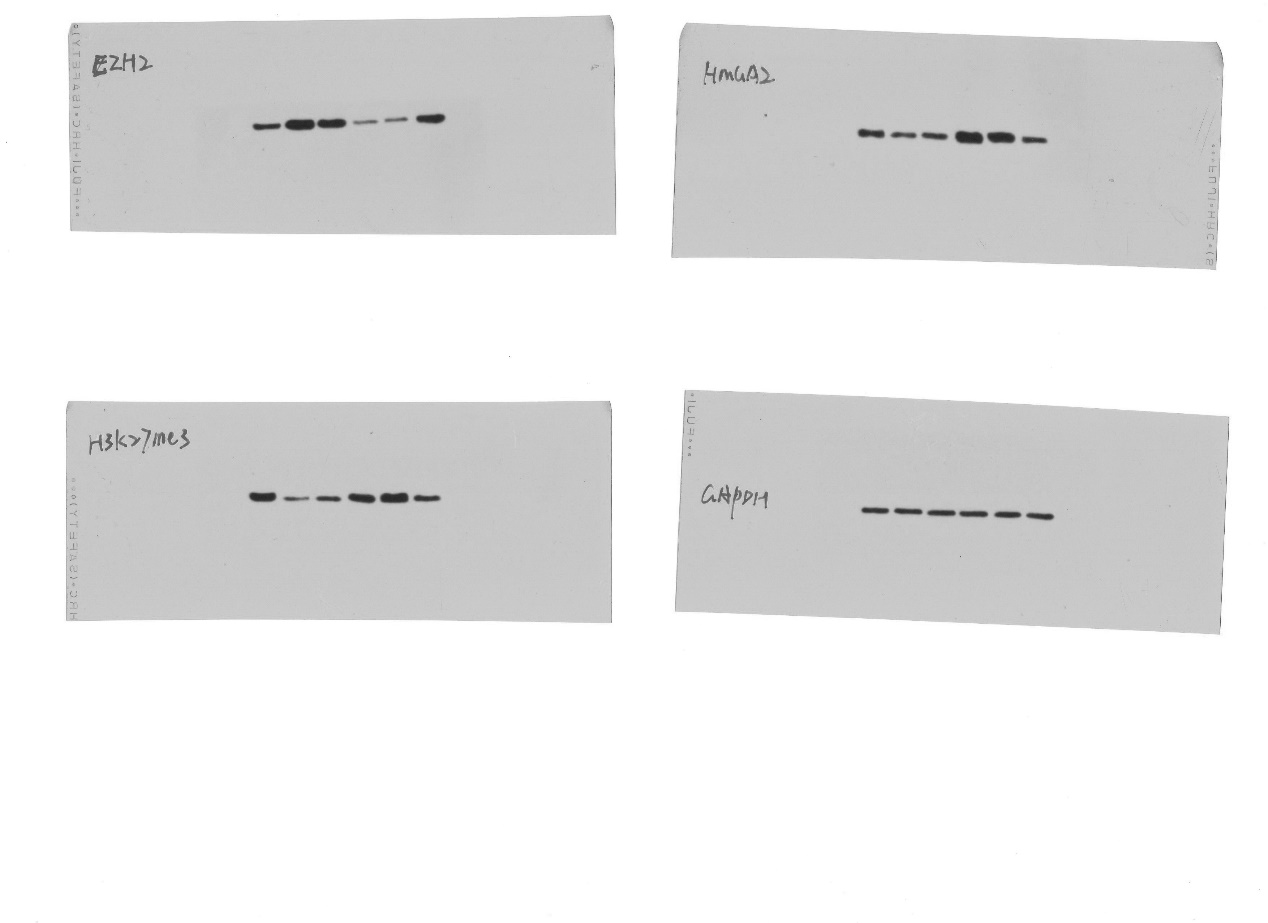
**

Supplement: Supplementary file 7 — Additional file 7: Original, full-length gel and lot images of Fig 7G. [file 12872_2022_2533_MOESM7_ESM.docx]
